# Evaluation of the Level of Zinc and Malondialdehyde in Basal Cell Carcinoma

**Published:** 2017-08

**Authors:** Ziba MAJIDI, Mahmoud DJALALI, Mohammad Hasan JAVANBAKHT, Mojtaba FATHI, Mahnaz ZAREI, Koorosh FOLADSAZ

**Affiliations:** 1. Dept. of Clinical Biochemistry, Faculty of Medicine, Zanjan University of Medical Sciences, Zanjan, Iran; 2. Dept. of Cellular & Molecular Nutrition, School of Nutritional Sciences & Dietetics, Tehran University of Medical Sciences, Tehran, Iran

**Keywords:** Basal cell carcinoma, Zinc, Malondialdehyde, Skin cancer

## Abstract

**Background:**

Basal Cell Carcinoma (BCC) is one of the most common skin cancers in the world and that use to lifestyle, increasing chemical pollutions, environmental factors and poor nutrition. The most important cause of this cancer is oxidative stress and free radicals so antioxidant activities for the body are so important. The aim of this study was to determine the variation of zinc and (Malondialdehyde) MDA in BCC patients.

**Methods:**

This study has been performed on case and control patients from 2013 to 2014. The samples were collected from cell carcinoma patients at Razi Hospital in Tehran, Iran. We evaluated the level of zinc with the use of Atomic Absorption Spectroscopy (AAS) method. Besides, we evaluated MDA with colorimetric assay.

**Results:**

The concentration of MDA was significantly higher in case group in comparison to control group (*P*=0.001). In addition, case group had lower concentration of zinc than the control group (*P*=0.000). There was no correlation between MDA and body mass index (BMI) and between zinc and BMI.

**Conclusion:**

All the patients with BCC showed a significant MDA serum in comparison with control group. However, significant decrease in zinc serum of the patients was seen that is because of consuming zinc during oxidative stress process so topical use of zinc in the form of 2+ ions could be effective on antioxidant protection against the sun UV radiation.

## Introduction

BCC is one of the most common malignancies among human neoplasms especially in peoples with light skins (1, 2). Around 80% of nonmelanoma skin cancers are associated with BCC (3). BCC is mostly seen in individuals from the puberty period until 35 yr of age (4). The incidence of diseases is highly related to the individuals’ ethnic as 90% of patients with Nevoid Basal Cell Carcinoma (NBCC) show BCC while the rate is about 40% in black patients (5). There are some factors, which are responsible for BCC, and among those UV, exposure is a major environmental melanomagenic agent (6). Besides, arsenic (7) alkalizing agents (8) chemical substances (9) and immunosuppression (10) are some other factors which are responsible for BCC. The best choice of treatment is surgical treatment. Along with that radiation therapy, liquid nitrogen cryo-therapy and ablative laser therapy, topical medical therapy, and systemic medical therapy are the other mentioned treatments for BCC (11). The invasive growth pattern of BCC may damage vessels, CNS, bone and cartilage, therefore, it is important to diagnose the disease at early stages (12).

Free radicals play an important role in several disease conditions such as diabetes mellitus, neurodegenerative disorders, cardiovascular diseases, respiratory diseases, cataract development, rheumatoid arthritis and in various cancers (13, 14). Oxidative stress is the result of high production rate of reactive oxygen species (ROS) and their low removal rate. Free radicals can cause damage to macromolecules such as nucleotides, proteins, carbohydrates and lipids. Moderate ROS overproduction can stimulate proliferation and survival of cancer cells (15, 16). Phospholipids in cell membrane are mostly composed of polyunsaturated fatty acids and consequently easily affected by free radicals (17). MDA is a final product of lipid peroxidation and many studies have used it as a marker of oxidative stress evaluation (18). Patients with BCC had a higher level of MDA than control group (19). MDA-derived epitopes are seen in skin in non-melanoma skin carcinoma tissue that exposed to UV (20). Serum MDA may serve as a screening test for malignant diseases at the early stages and for screening of the treatment progress in antioxidant therapy (21). In addition, the amount of serum MDA in individuals with squamous cell carcinoma (SCC) evaluated and showed the increased level of its (22).

Zinc is a ubiquitous trace element found throughout the body and plays an essential role in a multitude of cellular processes (23). The clinical features of zinc deficiency are growth retardation, testicular hypo-function, immune dysregulation, augmented oxidative stress and inflammation. Many studies have emphasized the effect of zinc as an antioxidant agent. Zinc acts as an antioxidant through various ways. Zinc shows two acute and chronic antioxidation mechanisms. Chronic mechanism suggests that exposure to zinc over long periods may induce other substances like metallothioneins which work as an antioxidant. The acute mechanism acts in two ways, protein sulphydryls conservation and decreasing the conversion of H_2_O_2_ to OH (24, 25). Zinc has been shown effective in preventing UV-induced damage and ameliorating malignancies (26). Two percent zinc sulfate solution could be useful in treatment of BCC (27). Reduction in the tissue and plasma zinc concentration is associated with prostate carcinoma (28). Patients with esophageal cancer had lower level of serum zinc (29). Skin BCC growth gradually but if not treated, may damage cartilage, bones, muscles and some other structures and can lead to death.

The current study was performed to determine the variation of zinc and MDA in BCC patients. Serum MDA level could be a useful test for prediction of BCC.

## Materials and Methods

This study has been performed on case and control patients from 2013 to 2014. The samples were collected from cell carcinoma patients at Razi Hospital in Tehran, Iran.

This study was approved by the Ethics Committee of Zanjan University of Medical Sciences; the consent form has been signed by all patients. The patients with other cancers, diabetes and blood pressure have been excluded from samples.

### 

#### Atomic Absorption Spectroscopy (AAS)

In this study, we have prepared 6% N-butyl alcohol solution (blanket dilution), which contains 60 cc of N-butyl alcohol that diluted to 1000 cc. In addition, four standard dilutions were prepared. To prepare standard solutions 9 gr NaCl and zinc (produced by Merck Company) has been added to blanket dilution. The standard solutions were prepared in 10, 20, 40 and 80 dilution.

The AAS devices (Younglin Company model 8020) were used to obtain the absorption curve of serum in compare with standard solutions. The absorption device set on this configure Wavelength: 213.9, electricity flow: 3 mA, Astelin gas flows: 1.8 L per second.

#### Lipid Peroxidation (MDA)

MDA kit made by Sigma-Aldrich Company with catalog number MAK085 used for this technique. To prepare this kit, vials need to be centrifuged before they are opened. To maintain reagent integrity, avoid repeated freeze/thaw cycles. Ultrapure water should be used for the preparation of all reagents. Then allow all components to come to room temperature before starting. Thiobarbituric acid (TBA) Solution – Reconstitute a bottle with 7.5 mL glacial acetic acid, then adjust the final volume to 25 mL with water. Sonication can be used to assist dissolution if necessary. Store at room temperature and use within 1 week of preparation. All samples and standards were run in duplicate.

### Statistical Analysis

SPSS ver. 22 (Chicago, IL, USA) (IBM Corp. Released 2013. IBM SPSS Statistics for Windows, Version 22.0 Armonk, NY: IBM Corp.) was used to analyze the data. To compare the means between case and control groups independent sample t-test was used. Pearson correlation, logistic regression, and linear regression were used to evaluate the correlation between variables.

## Results

Overall, 98 individuals have participated in this study, 40 cases, and 58 controls. 52.5% of participants were male and 46.5% were female. Demographic features of the participants including age; sex, BMI, and smoking status were shown in Table 1.

**Table 1:** Demographic features of the participants

**Variable**	**Amounts**	***P*-value**
**Subjects, No. (%) (n = 98)**		-
Case	40(40.8)	-
Control	58(59.2)	-
**Sex, No. (%)**			
Male	52(53.1)	-
Female	46(46.9)	-
**Age, mean ± SD (yr)**		0.000
Case	63.08 ± 10.45	-
Control	48.90 ± 8.69	-
BMI*, mean ± SD, kg/m 2		0.28**
Case	24.95 ± 3.79	-
Control	25.91± 4.60	-
**Smoking Status**		
Control smoker	6(10.3%)	-
Non-smoker	52(89.7%)	-
Case Smoker	14(35%)	-
Non-smoker	26(65%)	-

* BMI: body mass index

** *P*≤ 0.05 is significantly different

As shown in Table 2 the concentration of MDA was significantly higher in case group in comparison to control group (*P*=0.001). In addition, the case group had lower concentration of zinc than the control group (*P*=0.000). There was no correlation between BMI with zinc and MDA, also logistic regression showed no correlation between smoking with zinc and MDA.

**Table 2:** The comparison of amount of zinc and MDA in case and control groups

**Variable**	**MDA^b^, nmol/mL**	**Zinc, μg/dL**
Case	3.97 ± 1.65	78.65 ± 12.83
Control	2.91 ± 1.01	89.39 ± 12.47
*P*-Value^a^	0.001	0.000

a *P*-values less than 0.05 are significant.

b MDA: Malondialdehyde

## Discussion

BCC is one of the most common skin cancers in the world and is due to lifestyle, increasing chemical pollutions, environmental factors, and poor nutrition. This cancer is increasing in Iranian population. The most important cause of this cancer is oxidative stress and abnormal production of free radicals, thus the anti-oxidant activities of the body are so important (30, 31).

This case-control study was designed to evaluate the level of serum zinc and MDA to find association between antioxidant and BCC. To eliminate confounding factors, the groups matched in age, sex, and body mass index. The nutrient intakes were estimated by two days 24 h dietary recall. The groups were not statistically different in fat, carbohydrate, protein and energy intakes.

In the present study, MDA concentration as a peroxidation marker increased in BCC patients and zinc concentration as an antioxidant agent showed significant decrease in BCC patients.

This study showed that a significant relationship might exist between the decrease in zinc and BCC. Decrease in serum zinc of the patients may be because of consuming zinc during oxidative stress process. Zinc plays an anti-oxidant role (32) and acts against damage caused by UV radiation and is effective in wound healing and immune function. Topical use of zinc in the form of 2+ ions could be effective on antioxidant protection against the sun UV radiation (33, 34). The protective and antioxidant role of zinc showed that zinc acts as an antioxidant against the damaging effects of UV radiation in human fibroblast culture (35). Zinc consumption reduces peroxidation markers such as MDA (32). In other malignancies such as digestive problems, zinc reduced (36).

Oxidative stress can play different roles in pathogenesis of melanoma and non-melanoma skin cancers. The increased expression of the anti-oxidant in melanoma tissue was showed however it decreased in non-melanoma cancers. In addition, lipid peroxidation (LPO) significantly increased in melanoma cancer but in nonmelanoma cancer, this increase is not significant (37). UV exposure may reduce antioxidant capacity in non-melanoma cancers (38). Significant increase in MDA was found in squamous cell carcinoma that shows increased oxidative stress in tumors that may lead to mutation in DNA and cancer progression (39).

A significant relationship may exist between the increase in MDA and BCC. MDA is a highly toxic molecule produced from peroxidation of unsaturated fatty acids (40–43) and can change the biologic effect of proteins by changing their structure (44, 45). In addition, MDA is known as the most mutation cause of LPO products by reaction with DNA and produce Deoxyguanosine (46). MDA increases in stress oxidative condition. Increased MDA is sign of oxidation and exposure to adverse factors such as ultraviolet radiation or ozone (47).

## Conclusion

Oxidative stress can play important roles in the pathogenesis of many human cancers such as skin cancer. In BCC, decreased antioxidant components like zinc might contribute to multistep carcinogenesis.

## Ethical considerations

Ethical issues (Including plagiarism, informed consent, misconduct, data fabrication and/or falsification, double publication and/or submission, redundancy, etc.) have been completely observed by the authors.
